# Solvent-Free Microwave Extraction of *Thymus mastichina* Essential Oil: Influence on Their Chemical Composition and on the Antioxidant and Antimicrobial Activities

**DOI:** 10.3390/ph14080709

**Published:** 2021-07-22

**Authors:** André R. T. S. Araujo, Sandrine Périno, Xavier Fernandez, Cassandra Cunha, Márcio Rodrigues, Maximiano P. Ribeiro, Luisa Jordao, Lúcia A. Silva, Jesus Rodilla, Paula Coutinho, Farid Chemat

**Affiliations:** 1 Research Unit for Inland Development (UDI), Polytechnic Institute of Guarda, Av. Dr. Francisco Sá Carneiro, 50, 6300-559 Guarda, Portugal; cmpc7@hotmail.com (C.C.); mribeiro@ipg.pt (M.P.R.); 2 LAQV/REQUIMTE, Department of Chemical Sciences, Faculty of Pharmacy, University of Porto, Rua Jorge Viterbo Ferreira, 228, 4050-313 Porto, Portugal; 3 INRAE, UMR 408, GREEN Extraction Team, Avignon University, 84000 Avignon, France; sandrine.perino@univ-avignon.fr; 4 CNRS, ICN, Parc Valrose, Université Côte d’Azur, CEDEX 2, 06108 Nice, France; Xavier.FERNANDEZ@univ-cotedazur.fr; 5 CICS-UBI—Health Sciences Research Centre, University of Beira Interior, Av. Infante D. Henrique, 6200-506 Covilhã, Portugal; 6 Department of Environmental Health, Research and Development Unit, National Institute of Health Doutor Ricardo Jorge, Avenida Padre Cruz, 1649-016 Lisbon, Portugal; maria.jordao@insa.min-saude.pt; 7 Department of Chemistry, Faculty of Sciences, FibEnTech—Fiber Materials and Environmental Technologies, University of Beira Interior, Rua Marquês de Ávila e Bolama, 6201-001 Covilhã, Portugal; mlas@ubi.pt (L.A.S.); rodilla@ubi.pt (J.R.)

**Keywords:** essential oil, green extraction, large scale, solvent-free microwave extraction, *Thymus mastichina*

## Abstract

Solvent-free microwave extraction (SFME) is a combination of microwave heating and dry distillation performed at atmospheric pressure without the addition of water or organic solvents that has been proposed as a green method for the extraction of essential oils from aromatic and medicinal herbs. In this work, SFME and the conventional techniques of steam distillation (SD) and hydrodistillation (HD) were compared with respect to the extraction and antioxidant and antimicrobial activities of *Thymus mastichina* essential oil. The main constituent of essential oils obtained using different methods was 1,8-cineole (eucalyptol). The results showed that the essential oils extracted by means of SFME in 30 min were quantitatively (yield) and qualitatively (aromatic profile) similar to those obtained using conventional HD over 120 min. In addition, SFME generates less waste and less solvent, consumes less energy, and provides a higher yield for a shorter extraction time, which is advantageous for the extraction of the *T. mastichina* essential oil compared to SD. The antioxidant and antimicrobial activities of the *T. mastichina* essential oil obtained from either SFME or conventional extraction methods (SD or HD) showed a similar pattern. Large-scale experiments using this SFME procedure showed a potential industrial application.

## 1. Introduction

The *Lamiaceae* family is one of the largest and most distinctive families of lowering plants, with about 236 genera and almost 7200 species worldwide, and are best known for their unique essential oils [1]. In this family, the genus *Thymus* includes 214 species and 36 subspecies and is widely disseminated throughout the Mediterranean region with several species endemic to the Iberian Peninsula [2]. *Thymus mastichina* (white thyme) is an undershrub that has been used as a condiment/spice flavoring in seasoning traditional dishes and salads, to preserve olives, to aromatize olive oil, and as a substitute for salt [3,4]. In Portugal, white thyme can be found all over the country, except in calcareous regions [5]. It is an aromatic plant characterized by leaves arranged in opposite pairs and by zygomorphic and bilabiate flowers [6]. The essential oil isolated from the aerial parts of *T. mastichina* has been described for its antibacterial [7,8,9], antifungal [6,7,10] antioxidant [6,11,12] anti-inflammatory [6,11,13], and anti-Alzheimer activities [6,12], which are based on its specific chemical composition.

Conventional hydrodistillation (HD) is the most common method for the extraction of *T. mastichina* essential oil [5,6,7]. Moreover, microdistillation and solvent extraction techniques have also been employed [14]. However, some of these conventional extraction techniques have various disadvantages, including low extraction efficiency and the possibility of causing chemical modification of the oil components. These techniques often result in the loss of the most volatile molecules [15,16]. To overcome these shortcomings, The solvent-free microwave extraction (SFME) method has been recently developed and is one of the newest and promising techniques for essential oil extraction. SFME is based on the combination of microwave heating and distillation without adding water or organic solvents at atmospheric pressure [17,18,19]. This technique offers huge advantages, such as more effective heating, fast energy transfer, time-saving, low operating costs and is also considered an environmentally friendly “green technique, for the extraction of essential oils from plant materials” [20,21,22,23]. Taking into account the potential of this technique, previously validated, it was considered the need to proceed to the evaluation and characterization of the essential oils obtained by SFME from different maturation stage of plants as well as derived from fresh or dried plants. Thus, the present work aimed, for the first time to the best of our knowledge, to compare and characterize the yield and chemical composition of essential oils extracted from *T. mastichina* plants using conventional steam distillation (SD), HD, or SFME (on laboratory and pilot scales).

## 2. Results and Discussion

### 2.1. Yield and Quality of T. mastichina Essential Oil

The composition of essential oil from the flowers and aerial parts of *T. mastichina* obtained using SFME (laboratory and pilot scale) and conventional techniques (SD and HD) is summarized in Table 1. Globally, the composition of the essential oils extracted by the different methods is similar. The most representative compounds that were identified were monoterpenes, oxygenated monoterpenes, and sesquiterpenes. It should be noted that when the molecule is not quantified in the essential oil, it may be present at trace levels (compounds present at less than 0.1% in laboratory 1). From these components, the main volatile compound was 1,8-cineole (eucalyptol) and sabinene, α-pinene, β-pinene, linalool, and α-terpineol. Our results are consistent with previous reports from the literature, in which 1,8-cineole (also known as eucalyptol), linalool, camphor, α-pinene and camphene were identified as the major constituents by gas chromatography coupled with mass spectrometry detection (GC-MS) and gas chromatography with flame ionization detection (GC-FID) [6,7,10,13,24,25].

2,2-diphenyl-1-picrylhydrazyl (DPPH) scavenging activity was performed to verify if the antioxidant activity was preserved after the extraction process. The half maximal inhibitory concentration (IC_50_) values that were estimated were 1.69 and 2.44 mg equivalents of gallic acid/g for SD and SFME, respectively. The antioxidant activity was slightly higher using the SFME than when using the SD. The total phenolic content (TPC) quantified by the Folin–Ciocalteu reagent was 6.10^−3^ ± 5.10^−4^ and 6.10^−3^ ± 3.10^−4^ mg equivalents of gallic acid/g for SD and SFME, respectively. This could mean that its content is similar regardless of the extraction process and suggests that other compounds are responsible for the different antioxidant activities that were registered.

The *T. mastichina* essential oil obtained using the different extraction methods showed a broad spectrum of antimicrobial activity against several strains including the Gram-positive bacteria (methicillin-resistant *Staphylococcus aureus* (MRSA) ATCC 25923, methicillin-sensitive *Staphylococcus aureus* (MSSA) CIP 106760, and *Enterococcus faecalis* ATCC 29212), Gram-negative bacteria (*Escherichia coli* ATCC 25922 and *Pseudomonas aeruginosa* ATCC 27853), and yeast activity (*Candida albicans* ATCC 10231) (Table 2). The same minimum inhibitory concentration (MIC) values were achieved for the essential oils obtained using SD and SFME at the laboratory (ETHOS X) or pilot scale (Mac 75). In addition, it should be highlighted that the essential oil was equally active against MSSA and MRSA. In general, when comparing with the values reported from the literature, lower MIC values were obtained for the different species, except for the *Pseudomonas aeruginosa* ATCC 27853 [6,7,8,9,10].

There are three main chemotypes of essential oils isolated from *T. mastichina* collected in Portugal according to the major components 1,8-cineole, linalool, or 1,8-cineole/linalool. The most abundant essential oil distributed all over the country predominantly contains 1,8-cineole, as is the case for those from Freixedas in the Beira Interior region, while the other two components were only found in Estremadura, the oils from which are rich in linalool or 1,8-cineole/linalool [5,7,26].

Compared to previous analyses of *T. mastichina* essential oil from the Freixedas of Beira Interior (Planalto Dourado), the composition showed slight differences, in particular, the monoterpenes hydrocarbons were similar in the essential oils of the present study (10.79–20.59% vs. 18.65%) while showing higher levels of the oxygenated monoterpenes (68.95–80.2% vs. 67.71%) [26]. This suggests that the present essential oils were more valuable because oxygenated compounds are considered to be more odoriferous than monoterpene hydrocarbons.

**Figure 1:**
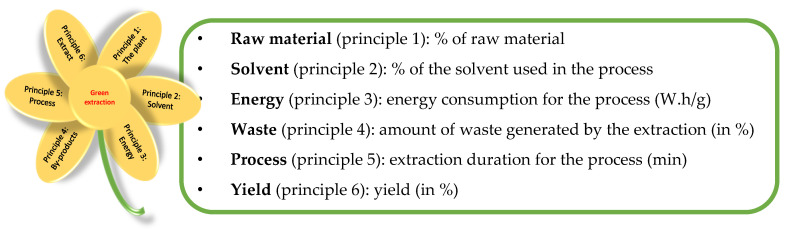
Description of the six principles of green extraction (raw material, solvent, energy, waste, process, and yield).

SD and SFME processes were evaluated according to the six principles of green extraction developed by Chemat et al. [27] (Figure 1). The following six parameters were chosen to compare both the SD and SFME processes in terms of solvent, energy consumption, raw material, process duration, yield, and waste in order to obtain the safest extract.

**Figure 2:**
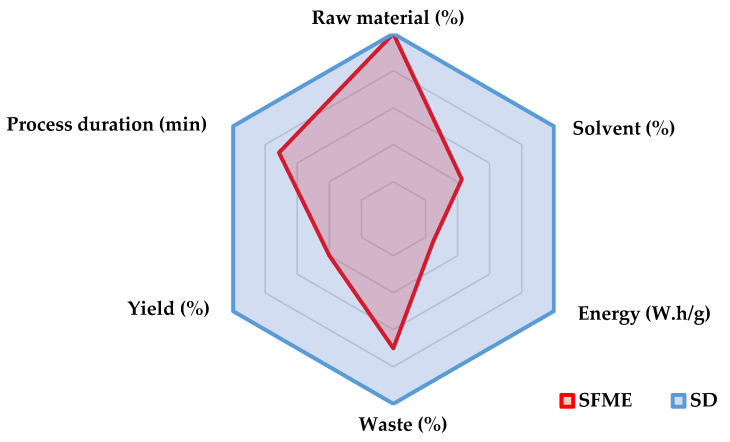
Steam distillation (SD) and solvent-free microwave extraction (SFME) processes classified according to the six principles of green extraction.

A graphical representation of the SD and SFME processes classified according to the six principles of green extraction is shown in Figure 2. For each principle, a value close to the center is a positive result. Conversely, a value far from the center is considered a negative result. Compared to SD, SFME generates less waste and less solvent, consumes less energy, and provides a higher yield over a shorter extraction time, which is advantageous for the extraction of *T. mastichina* essential oil.

### 2.2. Large Scale, Cost and Environmental Impact of T. mastichina Extraction

While conventional procedures such as SD or HD are often time and/or energy consuming, SFME provides numerous advantages from an industrial perspective. Microwave technology has shown wide-ranging commercial large-scale applications as a processing technology, with high returns on capital investment (with the break-even point of about 12 months). Microwave equipment is available from a laboratory (ETHOS X) to a pilot scale (Mac 75). The SFME could bring improvements in product efficiency, process enhancement, and sustainability, and low maintenance costs are achievable on a commercial scale.

In terms of extraction time, SFME method only requires 30 min of time at the laboratory scale and 60 min of time at the pilot scale without the addition of organic solvent or water, compared to conventional HD, which requires an extraction time of 120 min to heat the water and plant material to the extraction temperature followed by the evaporation of the water and essential oil. Relative to the yield of essential oils obtained using SFME at both the laboratory and pilot-scale (1.4–3.1%), the yield from SD (1.0–2.04%) and HD (3.16%) (Table 3) are quite similar. It also should be highlighted that better yields were obtained from dry plants that had been rehydrated when compared to fresh plants, even with SD and SFME obtained at the laboratory scale (ETHOS X).

The reduced extraction cost is advantageous for the proposed SFME method in terms of energy and time. The energy required to perform the two extraction methods is 176.5–227.8 W·h/g of essential oil for HD, 75.0–176.5 W·h/g of essential oil for SD, and 53.4–75.0 W·h/g of essential oil for SFME (laboratory and pilot-scale). The power consumption was determined using a Wattmeter at the microwave generator entrance and the electrical heater power supply. At the same time, the calculated quantity of the carbon dioxide released into the atmosphere was dramatically higher with HD (141.2–182.3 g CO_2_/g of essential oil) and SD (60.0–141.2 g CO_2_/g of essential oil) than with SFME (42.7–60.0 g CO_2_/g of essential oil). These calculations have been made according to calculation to obtain 1000 W.h from coal or fuel, from which 800 g of CO_2_ would be rejected into the atmosphere during the fossil fuel combustion [28].

Globally, considering these results, the laboratory and pilot study in a large-scale microwave reactor appears to be promising for the extraction of *T. mastichina* essential oil from the aerial parts. Thus, the important role and the potential of microwaves in the industry has begun to become evident.

## 3. Materials and Methods

### 3.1. T. mastichina Production and Harvest

Essential oils from *T. mastichina* were obtained from aerial parts from the cultivated plants grown in *Freixedas* (Beira Interior, Portugal), collected during the flowering phase (July 2018). The cultivated plants were collected from the Planalto Dourado farm. The collected aerial parts were composed of stems, leaves, and flowers. The aerial parts or only the flowers were used fresh in the different extraction assays. In some experiments, the aerial parts that had been dried at room temperature and stored to be protected from the light in dark bags over 3 months were used. Moisture content determination of fresh *T. mastichina* was conducted by dehydration in an electric oven at 80 °C. The average measured moisture content was 80.5 ± 0.5%.

### 3.2. Extraction Procedures

The extraction of the essential oil from *T. mastichina* was performed using conventional techniques (SD and HD) and SFME at both laboratory and pilot scales.

#### 3.2.1. Conventional Techniques: Hydrodistillation (HD) and Steam Distillation (SD) 

HD using the conventional technique: 500 g of dry *T. mastichina* were rehydrated and distilled using a Clevenger-type apparatus and according to the European Pharmacopeia and were then extracted with 10 L of water for 120 min (until no more essential oil was obtained). The essential oil was collected, dried with anhydrous sodium sulfate, and stored at 4 °C until use.

SD using the conventional technique: 500 g of fresh or dry (previously rehydrated) *T. mastichina* aerial parts and flowers were steamed in 3 L of water and were then extracted by applying 1800 Watt for 60 min (until no more essential oil was obtained). The essential oil was collected, dried with anhydrous sodium sulfate, and stored at 4 °C until use.

#### 3.2.2. Solvent-Free Microwave Extraction (SFME): Laboratory and Pilot Scale

SFME was performed in a laboratory microwave oven ETHOS X oven (Milestone, Italy). During experiments, time, temperature, pressure, and power were controlled by the software. The experiment was conducted at atmospheric pressure with 500 g of fresh or dry (previously rehydrated with 1.8 L of water during 30 min) *T. mastichina* aerial parts and flowers at 1800 W. A cooling system outside of the microwave cavity continuously condensed the distillate using a Clevenger-type apparatus. Condensed water was returned to the flask and heating was continued at 100 °C until no more essential oil was obtained. The essential oil was collected, dried under anhydrous sodium sulfate, and stored at 4 °C until subsequent analysis.

The Mac 75 apparatus (multimode microwave reactor) containing four magnetrons (4 × 1500 W, 2450 MHz) with a maximum power of 6000 W was used to obtain *T. mastichina* essential oil at a pilot scale. The experiment was conducted with 4000 g of dry (previously rehydrated) *T. mastichina* aerial parts that had been soaked in water and applying a power of 6000 W for 60 min. Similarly, the essential oil was collected, dried under anhydrous sodium sulphate, and stored at 4 °C until subsequent analysis.

### 3.3. Chemical Analysis of Essential Oils Compounds by Gas Chromatography with Flame Ionization Detection (GC-FID) and Mass Spectrometry (GC-MS)

Two independent laboratories determined the chemical analysis of essential oil compounds in order to better characterize their composition.

#### 3.3.1. Chromatographic Method (Independent Laboratory 1)

A 20% essential oil solution in dichloromethane was prepared for GC analysis. A GC-FID quantitative analysis of volatile compounds was conducted using an Agilent 6850 gas chromatograph equipped with an Equity-5 column (length 15 m × 0.1 mm i.d., film thickness 100 µm) and an FID detector. The analyses were performed by injecting 0.2 µL of sample at a split ratio of 800:1. The oven temperature was programmed starting at 40 °C for 2 min, 5 °C/min up to 270 °C, and then 270 °C for 2 min. In parallel, gas chromatography coupled with mass spectrometry detection (GC-MS) analysis was conducted using an Agilent 6890 N coupled to an Agilent 5973 MS (Agilent, Massy, France). Samples were analyzed on a fused-silica capillary column HP-5MS 5% Phenyl Methyl Siloxane (length 30 m × 0.25 mm i.d., film thickness 250 µm). The analyses were performed by injecting 0.1 µL of sample at a split ratio of 50:1. The oven temperature was programmed starting at 40 °C, 2 °C/min up to 270 °C. For the identification of the compounds, commercial databases (Nist 98 and Wiley) and comparison of spectra with laboratory mass spectra libraries built up from pure substances and MS literature data were used [29]. Identification of the components was also based on their GC retention indices on an apolar column, using the homologous series of n-alkanes (C_5_–C_26_) as a reference, and their comparison with those of literature data [30,31]. Relative amounts of individual components were based on peak areas obtained without FID response factor correction. Three replicates were performed for each sample.

#### 3.3.2. Chromatographic Method (Independent Laboratory 2)

Samples for analysis had been prepared from the different essential oils from each process by dilution to 20% in dichloromethane. The analysis of the components of each sample and the quantification was performed on a GC-FID from Agilent Mod. 6850. The chromatography column used was an Equity (length 15 m × 0.1 mm id, film thickness 100 µm). Each analysis was performed by injecting 0.2 µL of sample at a split ratio 800:1. The oven temperature was programmed starting at 40 °C for 2 min, increasing from 5 °C/min to 270 °C and ending at 270 °C for 2 min. In parallel, GC-MS was performed to identify the components of each essential oil. It was conducted using an Agilent 6890 N coupled to an Agilent 5973 MS detector (Agilent, Massy, France). Samples were analyzed on a 5% HP-5MS fused-silica capillary column of phenylmethylsiloxane (length 30 m × 0.25 mm ID, film thickness 250 µm). The analyses were performed by injecting 0.1 µL of sample at a split ratio of 50:1. The oven temperature was programmed starting at 40 °C, with a temperature increase of 2 °C/min to 270 °C. The identification of the compounds and the quantification analysis were performed in a similar way to independent laboratory 1.

### 3.4. Antioxidant Assays

The antioxidant capacity of the obtained essential oils was evaluated using two methods: DPPH and TPC.

#### 3.4.1. Free Radical Scavenging Activity

Free radical scavenging activity was determined by the DPPH free radical. The method is based on the reduction of DPPH free radicals by the essential oil antioxidants. 25 mg of DPPH was solubilized in 100 mL of methanol and diluted to 1:10 with methanol. This was based on the Brand-Williams modified procedure [32]. Different solutions of *T. mastichina* essential oil were prepared with methanol at four concentrations: 12.5; 25; 50; and 100 mg/mL. A 50 μL volume of sample volume was mixed with 2 mL of DPPH solution and incubated at a temperature of 22 °C for 40 min while being sheltered from light. The absorbances were measured at 517 nm by using a UV−vis spectrophotometer (UV-1800, Shimadzu, Japan). The absorbances were then converted into a percentage of the antioxidant activity (%) using the following equation:

$$\mathrm{Antioxidant}\mathrm{activity}\;(\%)=100-\left(\frac{sample\;absorbance-blank\;absorbance}{reference\;absorbance}\times 100\right)$$

where *sample absorbance* is the absorbance of the sample at a given concentration, *blank absorbance* is the absorbance of the pure solvent (methanol), and *reference absorbance* is the absorbance of the DPPH solution.

The determination of the IC_50_ that corresponded to 50% was based on the linear equation curves of the essential oil concentrations. The DPPH scavenging capacity of the essential oil was expressed in mg gallic acid equivalent per gram.

#### 3.4.2. Total Polyphenol Analysis

The TPC was determined using the Folin–Ciocalteu method [33] with some minor modifications. First, 50 μL of essential oil filtered on 0.45 μm was mixed with 1250 μL of a 5-fold diluted Folin–Ciocalteu reagent in water. The solutions were mixed thoroughly and incubated at room temperature (22 °C) for 1 min. A 1 mL volume of 10% sodium carbonate (Na_2_CO_3_) was then added to the solution and mixed thoroughly. Solutions were incubated at room temperature (22 °C) for 30 min and were sheltered from light. Absorbances were measured at 750 nm using an ultraviolet–visible (UV−Vis) spectrophotometer (UV-1800, Shimadzu, Japan). Standardization curves were conducted with solutions at different concentrations. The TPC was expressed as milligrams of gallic acid equivalent per gram. The presented data results from triplicate analysis.

### 3.5. Antimicrobial Activity

#### 3.5.1. Microbial Strains

The antimicrobial activity of the essential oils against a representative group of human pathogens including Gram-negative (*Escherichia coli* ATCC 25922, *Pseudomonas aeruginosa* ATCC 27853) and Gram-positive (*Staphylococcus aureus* ATCC 25923, *Staphylococcus aureus* CIP 106760, *Enterococcus faecalis* ATCC 29212) Enteroacteriaceae species and the yeast *Candida albicans* ATCC 10231 was evaluated. The microorganisms were stored in tryptic soy broth supplemented with 20% (*v*/*v*) glycerol at—80 °C. In all assays, fresh overnight cultures were prepared on Mueller Hinton agar or Sabouraud with chloramphenicol agar for bacteria or yeast, respectively.

#### 3.5.2. Determination of Minimum Inhibitory Concentration (MIC)

The MIC corresponds to the lowest concentration of essential oil that inhibits the development of a certain microorganism. The broth microdilution method was used to determine the MIC of the essential oils adapted from the Clinical Laboratory Standards Institute guidelines. The 0.5% solution of essential oil in absolute ethanol (*v*/*v*) was freshly prepared and serially diluted in culture medium (1:1) over the concentration range of 0.25–0.00024% (*v*/*v*). The culture medium was used as a negative/sterility control, the culture medium inoculated with the microorganisms was used as positive/growth control, and the absolute ethanol diluted in culture medium over the concentration range of 0.25–0.00024% (*v*/*v*) inoculated with the microorganisms was used as a solvent control. The inoculum was prepared from overnight cultures of each microorganism with final concentrations adjusted to 10^5^ cells/mL. Mueller Hinton or Sabouraud broth were used for bacteria and *Candida*, respectively. In all cases, the incubation was performed at 37 °C for 24 h, as the MIC is defined as the lowest concentration of the essential oil at which the microorganism does not demonstrate visible growth during this incubation period. At least two independent experiments conducted in duplicate were performed.

## 4. Conclusions

*T. mastichina* essential oil obtained by either conventional extraction methods (SD or HD) or the SFME present a similar chemical composition and antioxidant and antimicrobial capacity. SFME is quicker, more effective, and more environmentally friendly and thus proved to be an outstanding alternative offering significant advantages over traditional extraction methods (SD and HD). Furthermore, this study shows potential applicability of SFME in the food, cosmetic, and pharmaceutical industries (at laboratory and pilot scale) while preserving the *T. mastichina* essential oil antioxidant and antimicrobial properties against relevant human pathogens.

## Figures and Tables

**Table 1:** Chemical compositions of *Thymus mastichina* essential oils obtained from steam distillation (SD), hydrodistillation (HD), or solvent-free microwave extraction (SFME) at laboratory (ETHOS X) or pilot scale (Mac 75) from flowers and fresh or dry aerial parts.

Identification	Molecular Formula	Retention Indice (Lab 1)	Retention Indice (Lab 2)	Literature Retention Index	%SD Flower (Lab 2)	%ETHOS X Flower (Lab 2)	%SD Fresh Aerial Parts (Lab 1)	%SD Fresh Aerial Parts (Lab 2)	%ETHOS X Fresh Aerial Parts (Lab 1)	%ETHOS X Fresh Aerial Parts (Lab 2)	%HD Dry Aerial Parts Rehydrated (Lab 1)	%HD Dry Aerial Parts Rehydrated (Lab 2)	%SD Dry Aerial Parts Rehydrated (Lab 1)	%SD Dry Aerial Parts Rehydrated (Lab 2)	%ETHOS X Dry Aerial Parts Rehydrated (Lab 1)	%ETHOS X Dry Aerial Parts Rehydrated (Lab 2)	%Mac 75 Dry Aerial Parts Rehydrated (Lab 1)	%Mac 75 Dry Aerial Parts Rehydrated (Lab 2)
**Monoterpenes**																		
Tricyclene	C_10_H_16_	914	914	920	-	-	t	0.02	t	0.02	t	-	t	0.02	t	0.01	t	0.02
α-Thujene	C_10_H_16_	920	924	924	0.20	0.21	0.12	0.15	0.14	0.18	0.19	0.20	0.25	0.29	0.20	0.24	0.26	0.29
α-Pinene	C_10_H_16_	927	927	932	3.21	3.41	3.27	3.71	3.63	4.35	3.32	3.31	4.15	4.47	3.31	3.71	4.15	4.39
Camphene	C_10_H_16_	938	938	946	0.21	0.23	0.26	0.30	0.28	0.32	0.28	0.27	0.34	0.35	0.27	0.29	0.30	0.31
Sabinene	C_10_H_16_	968	968	968	3.17	3.61	4.45	4.39	4.64	4.98	4.62	3.37	5.21	4.44	4.39	3.90	5.09	4.27
β-Pinene	C_10_H_16_	974		974	4.55	4.87	-	5.23	-	5.83	-	4.82	-	6.20	-	5.22	-	6.02
3-Octanone	C_8_H_16_O	979		979	-	-	-	-	-	0.01	-	-	-	-	-	0.01	-	0.02
2,3-Dehydro-1,8-cineole	C_10_H_16_O	976		978	-	-	t	-	t		t	-	t	-	t	-	t	-
Myrcene	C_10_H_16_	983	983	983	1.40	1.57	1.94	1.72	2.03	2.00	1.72	1.46	2.26	2.04	1.83	1.76	2.35	2.10
3-Octanol	C_8_H_18_O	988		988	-	0.02	-	-	-	0.02	-	-	-	0.02	-	0.02	-	0.02
α-Phellandrene	C_10_H_16_	997	997	997	0.05	0.04	t	0.05	t	0.05	t	0.04	t	0.06	t	0.05	t	0.05
α-Terpinene	C_10_H_16_	1008	1008	1009	0.20	0.17	t	0.17	t	-	t	0.22	0.11	0.31	t	0.21	t	0.33
Cymene *	C_10_H_14_	1015	1015	1014	1.12	0.85	-	0.79	t	0.85	t	0.95	-	0.89	t	0.95	t	0.78
1,8- cineole (eucalyptol)	C_10_H_18_O	1028	1028	1022	62.53	59.79	67.89	63.40	66.28	52.01	68.46	60.92	70.60	63.63	67.41	55.68	64.99	56.30
(Z)-β-Ocimene	C_10_H_16_	1032		1032	0.05	0.04	0.25	0.04	0.26	0.04	0.20	0.04	0.26	0.05	0.20	0.03	0.25	0.05
(E)-β-Ocimene	C_10_H_16_	1039	1039	1037	0.16	0.20	-	0.30	-	0.36	-	0.21	-	0.28	-	0.23	-	0.28
γ-Terpinene	C_10_H_16_	1049	1049	1051	0.51	0.65	0.52	0.83	0.58	0.99	0.65	0.77	0.82	1.04	0.59	0.73	0.82	1.10
(E)-Sabinene hydrate	C_12_H_20_O_2_	1055	1055	1058	0.43	0.66	0.55	0.54	0.43	0.60	0.48	0.49	0.35	0.38	0.62	0.72	0.30	0.33
Linalool oxide *	C_10_H_18_O_2_	1058	1058	1076	-	0.04	t	0.03	t	0.03	t	0.03	t	0.02	t	0.04	t	0.02
Terpinolene	C_10_H_16_	1077	1077	1080	0.10	0.12	t	0.11	t	0.14	t	0.12	0.11	0.15	t	0.13	0.12	0.18
(Z)-Sabinene Hydrate	C_10_H_18_O	1085	1055	1080	-	-	t	-	t	-	0.15	-	t	-	0.15		t	-
Linalool	C_10_H_18_O	1091	1091	1086	3.19	4.15	3.64	3.61	3.51	4.24	3.56	3.97	3.19	3.70	3.73	4.39	3.98	4.44
Hotrienol	C_10_H_16_O	1101		1101	-	0.06	-	0.04	-	0.05	-	0.05	-	0.04	-	0.04	-	0.06
p-Menth-2-en-1-ol	C_10_H_18_O	1105	1105	1108	0.05	0.05	t	0.04	t	0.05	-	0.06	t	0.03	-	0.05	-	0.05
α-Campholene aldehyde	C_10_H_16_O	1111		1111	-	0.02	-	0.02	-	0.03	-	-	-	0.02	-	0.03	-	0.02
Pinocarveol	C_10_H_16_O	1123	1123	1133	0.10	0.08	t	0.07	t	0.10	t	0.12	t	0.07	t	0.10	t	-
Camphor	C_10_H_16_O	1126	1126	1140	0.07	0.11	t	0.05	t	0.06	t	0.09	t	0.04	t	0.06	t	0.10
Pinocarvone	C_10_H_14_O	1138	1138	1140	0.04	-	t	0.04	t	0.05	t	0.05	t	0.03	t	0.05	t	0.02
Borneol	C_10_H_18_O	1144		1154	-	-	t	-	t	-	t	-	t	-	t	-	t	-
Terpineol *	C_10_H_18_O	1152	1152	1160	2.45	2.40	2.00	2.27	1.82	2.64	2.13	2.69	1.09	1.51	2.03	2.70	1.60	2.06
Terpinen-4-ol	C_10_H_18_O	1173	1173	1165	0.63	0.52	t	0.46	t	0.58	t	0.77	t	0.46	t	0.55	t	0.70
α-Terpineol	C_10_H_18_O	1179	1179	1175	6.33	5.98	4.50	5.31	4.07	6.07	4.60	6.20	2.31	3.36	4.51	6.20	3.68	4.97
(Z)-Dihydrocarvone	C_10_H_16_O	1183	1183	1181	0.29	0.25	t	0.21	t	0.25	t	0.29	t	0.15	t	0.27	t	0.20
(E)-Dihydrocarvone	C_10_H_16_O	1217	1217	1190	0.20	0.18	t	0.18	t	0.22	t	0.18	t	0.09	t	0.18	t	0.13
Isobornyl formate	C_11_H_18_O_2_	1218		1228	-	-	t	-	t	-	t	-	-	-	t	-	-	-
Piperitol	C_10_H_18_O	1207		1207	-	-	-	-	-	-	-	-	-	-	-	0.02	-	0.01
trans-Carveol	C_10_H_16_O	1215		1215	-	0.02	-	-	-	0.02	-	0.02	-	-	-	0.03	-	-
Bornyl or isobornyl acetate	C_12_H_20_O_2_	1268	1268	1275	0.04	0.03	t	0.02	t	0.03	t	0.04	t	0.03	t	0.03	t	0.02
Carvacrol methyl ether	C_11_H_16_O	1241		1241	0.56	0.53	-	0.49	-	0.62	-	0.60	-	0.47	-	0.61	-	0.60
Linalool acetate	C_12_H_20_O_2_	1254		1254	-	0.02	-	0.03	-	0.04	-	0.02	-	0.02	-	0.02	-	0.02
Endobornyl acetate	C_12_H_20_O_2_		1272	1284	0.08	0.02	-	0.02	-	0.01	-	0.04	-	0.02	-	0.02	-	0.02
Carvacrol	C_10_H_14_O	1298		1298	1.30	1.19	-	-	-	1.16	-	1.26	-	0.57	-	1.31	-	0.91
Thymol	C_10_H_14_O	1293	1293	1287	-	0.05	0.76		0.61	0.04	0.82	-	0.37	-	0.78	0.05	0.59	0.02
Sesquiterpenes																		
Bicycloelemene	C_15_H_24_	1330		1330	-	0.03	-	0.02	-	0.04	-	0.03	-	0.02	-	0.02	-	0.02
α-Copaene	C_15_H_24_	1371	1347	1347	-	0.02	t	-	t	0.02	t	-	t	0.02	t	0.02	t	0.02
Bourbonene *	C_15_H_24_	1379	1379	1375	0.21	0.19	t	0.11	0.15	0.23	0.10	0.13	0.11	0.15	0.15	0.21	0.15	0.21
β-Elemene	C_15_H_24_	1385	1385	1402	0.08	0.09	t	0.06	t	0.13	t	0.06	t	0.07	t	0.10	t	0.12
α-Gurjunene	C_15_H_24_	1404	1404	1420	0.03	0.03	t	-	t	0.04	t	0.02	t	0.03	t	0.04	t	0.06
β-Caryophyllene	C_15_H_24_	1413	1413	1417	0.33	0.33	0.20	0.20	0.35	0.49	0.16	0.18	0.19	0.25	0.29	0.39	0.36	0.45
Germacrene D	C_15_H_24_			1484	0.04	0.04	-	0.02	-	0.05	-	0.02	-	0.03	-	0.05	-	0.05
Aromadendrene	C_15_H_24_	1448	1448	1446	-	-	t	-	t	0.02	t	0.02	t	0.02	t	0.04	t	0.05
β-Farnesene	C_15_H_24_	1440		1440	0.04	0.04	-	0.02	-	0.04	-	0.02	-	0.02	-	0.04	-	0.05
α-Bisabolene	C_15_H_24_	1505	1501	1505	0.06	0.07	-	0.03	-	0.08	-	0.04	-	0.04	-	0.08	-	0.09
4,5-Dehydro-isolongifolene	C_15_H_22_	1544		1544	-	0.03	-	-	-	-	-	0.02	-	-	-	0.03	-	-
allo-Aromadendrene	C_15_H_24_	1454	1464	1460	0.10	0.10	t	0.06	t	0.16	t	0.06	t	0.07	t	0.12	t	0.05
Aromadendrene isomer	C_15_H_24_			1458	0.06	0.06	-	0.02	-	0.07	-	0.03	-	0.04	-	0.09	-	-
β-cubebene	C_15_H_24_	1387		1387	0.82	0.93	-	0.64	-	1.54	-	0.49	-	0.63	-	1.08	-	1.24
β-selinene	C_15_H_24_	1489		1489	-	-	-	-	-	0.03	-	0.02	-	-	-	0.04	-	0.04
Eremophilene	C_15_H_24_	1486		1486	0.07	-	-	-	-	0.09	-	-	-	-	-	0.08	-	0.09
Bicyclogermacrene	C_15_H_24_	1500		1500	1.90	2.15	-	1.92	-	3.81	-	1.35	-	-	-	2.47	-	2.88
Valencene	C_15_H_24_	1489		1485	-	-	1.45	-	2.29	-	0.85	-	0.84	-	1.62	-	1.96	-
α-Bisabolene	C_15_H_24_	1501		1496	-	-	0.36	-	0.71	0.08	0.28	-	0.36	-	0.64	-	0.80	-
β -Bisabolene	C_15_H_24_	1505	1501	1505	1.32	1.31	-	0.56	-	1.50	-	0.61	-	0.78	-	1.46	-	1.56
γ -Cadinene	C_15_H_24_	1513		1513	0.05	0.05	-	0.02	-	0.04	-	0.03	-	0.03	-	0.06	-	0.07
δ-Cadinene	C_15_H_24_	1513	1513	1516	0.12	0.11	t	0.05	t	0.12	t	0.06	t	0.07	t	0.15	t	0.17
Spathulenol	C_15_H_24_O	1569	1569	1567	0.35	0.38	t	0.09	t	0.22	t	0.57	t	0.19	t	0.46	t	0.24
Caryophyllene oxide	C_15_H_24_O	1582		1582	0.10	0.11	-	0.05	-	0.10	-	0.18	-	0.05	-	0.13	-	0.07
Viridiflorol	C_15_H_26_O	1592		1592	0.44	0.52	-	0.32	-	0.59	-	0.88	-	0.29	-	0.69	-	0.35
10-Epi-γ-eudesmol	C_15_H_26_O	1622		1622	-	0.02	-	-	-	0.02	-	0.04	-	-	-	0.03	-	-
Isoespathulenol	C_15_H_24_O			1640	0.04	0.07	-	-	-	0.06	-	-	-	0.03	-	0.09	-	-
τ-Muurolol	C_15_H_26_O	1641		1641	-	-	-	-	-	0.05	-	0.08	-	-	-	0.07	-	-
Juniper camphor	C_15_H_26_O	1691		1691	0.40	0.58	-	0.23	-	0.53	-	0.86	-	0.29	-	0.70	-	0.32
% of identified compounds					99.78	99.4	92.16	99.06	91.78	99.19	92.57	99.37	92.92	98.37	92.72	99.38	91.75	98.65
% of monoterpenes among the identified compounds					14.93	15.97	10.81	17.81	11.56	20.11	10.98	15.78	13.51	20.59	10.79	17.46	13.34	19.41
% of oxygenated monoterpenes among the identified compounds					78.29	76.17	79.34	76.83	76.72	68.93	80.2	77.79	77.91	74.66	79.23	73.18	75.14	71.04
% of sesquiterpenes among the identified compounds					6.56	7.26	2.01	4.42	3.5	10.15	1.39	5.8	1.5	3.12	2.7	8.74	3.27	8.2

* Isomer not characterized, *Method 1: t*: traces (compounds present at less than 0.1%).

**Table 2:** Evaluation of the antimicrobial activity of *Thymus* mastichina essential oils obtained using steam distillation (SD) and solvent-free microwave extraction (SFME) at laboratory (ETHOS X) and pilot scale (Mac 75).

Microorganism	Minimum Inhibitory Concentration (MIC) (% (*v*/*v*))		
	SD	ETHOS X	Mac 75
*Staphylococcus aureus* (ATCC 25923) *	0.031	0.031	0.031
*Staphylococcus aureus* (CIP 106760) **	0.031	0.031	0.031
*Enterococcus faecalis* (ATCC 29212)	0.031	0.031	0.031
*Escherichia coli* (ATCC 25922)	0.156	0.156	0.156
*Pseudomonas aeruginosa* (ATCC 27853)	0.015	0.015	0.015
*Candida albicans* (ATCC 10231)	0.078	0.078	0.078

* Methicillin resistant *S. aureus* (MRSA) and ** methicillin susceptible *S. aureus* (MSSA).

**Table 3:** Extraction time, yield, energy consumption, and environmental impact of *Thymus mastichina* essential oils obtained using steam distillation (SD), hydrodistillation (HD), and solvent-free microwave extraction (SFME) at laboratory (ETHOS X) and pilot scale (Mac 75) with the use of flowers and fresh and dry aerial parts.

Identification	%SD Flower	%ETHOS X Flower	%SD Fresh Aerial Parts	%ETHOS X Fresh Aerial Parts	%HD Dry Aerial Parts Rehydrated	%SD Dry Aerial Parts Rehydrated	%ETHOS X Dry Aerial Parts Rehydrated	%Mac 75 Dry Aerial Parts Rehydrated
Extraction time (min)	60	30	30	30	120	60	30	60
Yield (%)	0.91	3.1	1	1.4	3.16	2.04	2.4	2.81
Energy consumption (W.h/g of essential oil)	395.6	58.1	75.0	53.6	227.8	176.5	75.0	53.4
Environmental impact (g CO_2_/g essential oil)	316.5	46.5	60.0	42.9	182.3	141.2	60.0	42.7

## Data Availability

Not applicable.
